# Integration of macromolecular complex data into the *Saccharomyces* Genome Database

**DOI:** 10.1093/database/baz008

**Published:** 2019-02-04

**Authors:** Edith D Wong, Marek S Skrzypek, Shuai Weng, Gail Binkley, Birgit H M Meldal, Livia Perfetto, Sandra E Orchard, Stacia R Engel, J Michael Cherry, the SGD Project

**Affiliations:** 1 Department of Genetics, Stanford University, Porter Drive, Palo Alto, CA, USA; 2 European Molecular Biology Laboratory, European Bioinformatics Institute (EMBL-EBI) Wellcome Genome Campus, Hinxton, Cambridge, UK

## Abstract

Proteins seldom function individually. Instead, they interact with other proteins or nucleic acids to form stable macromolecular complexes that play key roles in important cellular processes and pathways. One of the goals of *Saccharomyces* Genome Database (SGD; www.yeastgenome.org) is to provide a complete picture of budding yeast biological processes. To this end, we have collaborated with the Molecular Interactions team that provides the Complex Portal database at EMBL-EBI to manually curate the complete yeast complexome. These data, from a total of 589 complexes, were previously available only in SGD’s YeastMine data warehouse (yeastmine.yeastgenome.org) and the Complex Portal (www.ebi.ac.uk/complexportal). We have now incorporated these macromolecular complex data into the SGD core database and designed complex-specific reports to make these data easily available to researchers. These web pages contain referenced summaries focused on the composition and function of individual complexes. In addition, detailed information about how subunits interact within the complex, their stoichiometry and the physical structure are displayed when such information is available. Finally, we generate network diagrams displaying subunits and Gene Ontology annotations that are shared between complexes. Information on macromolecular complexes will continue to be updated in collaboration with the Complex Portal team and curated as more data become available.

## Introduction

Cellular processes are dynamic and highly organized, both in time and space, and individual proteins rarely work in isolation; they frequently have to remain tightly bound to other proteins or small molecules in order to perform specific cellular functions and to carry out their roles within cellular pathways. Having knowledge about such protein complexes is essential to our understanding of biology. Since its inception, one of the goals of *Saccharomyces* Genome Database (SGD; www.yeastgenome.org) has been to facilitate research by providing comprehensive knowledge about budding yeast cell biology (1). Beginning with the annotation of the *Saccharomyces cerevisiae* genome sequence, SGD has long curated information to specific loci within the genome. Currently, a variety of data is available through SGD, including mutant phenotypes, gene expression and genetic interactions of various loci, to name a few. To further expand the knowledge of cellular processes available in our database, we sought to extend curation from single loci to macromolecular complexes. We collaborated with the Complex Portal (www.ebi.ac.uk/complexportal) to curate the yeast macromolecular complexome. This set of complexes was chosen based on published literature and previous curation of subunits to Gene Ontology (GO) macromolecular complex terms. When biocurators encountered literature about novel complexes, these were added to the list and curated as well. Using the shared Complex Portal curation tool, our groups collaborated to curate 589 macromolecular yeast complexes (2, 3). We have integrated these data into SGD and now provide Complex summary pages available for researchers visiting the SGD website.

## Curation of macromolecular complexes

SGD’s decision to curate macromolecular complexes coincided with the ongoing work of the Complex Portal curation group, which curates binary protein–protein interactions in the IntAct database (4) as well as macromolecular complexes from multiple organisms. Establishing a collaboration with the Complex Portal had the following advantages: use of an established curation interface and the same curation standards, no redundancy and data synchronization between the two groups.

**Figure 1:**
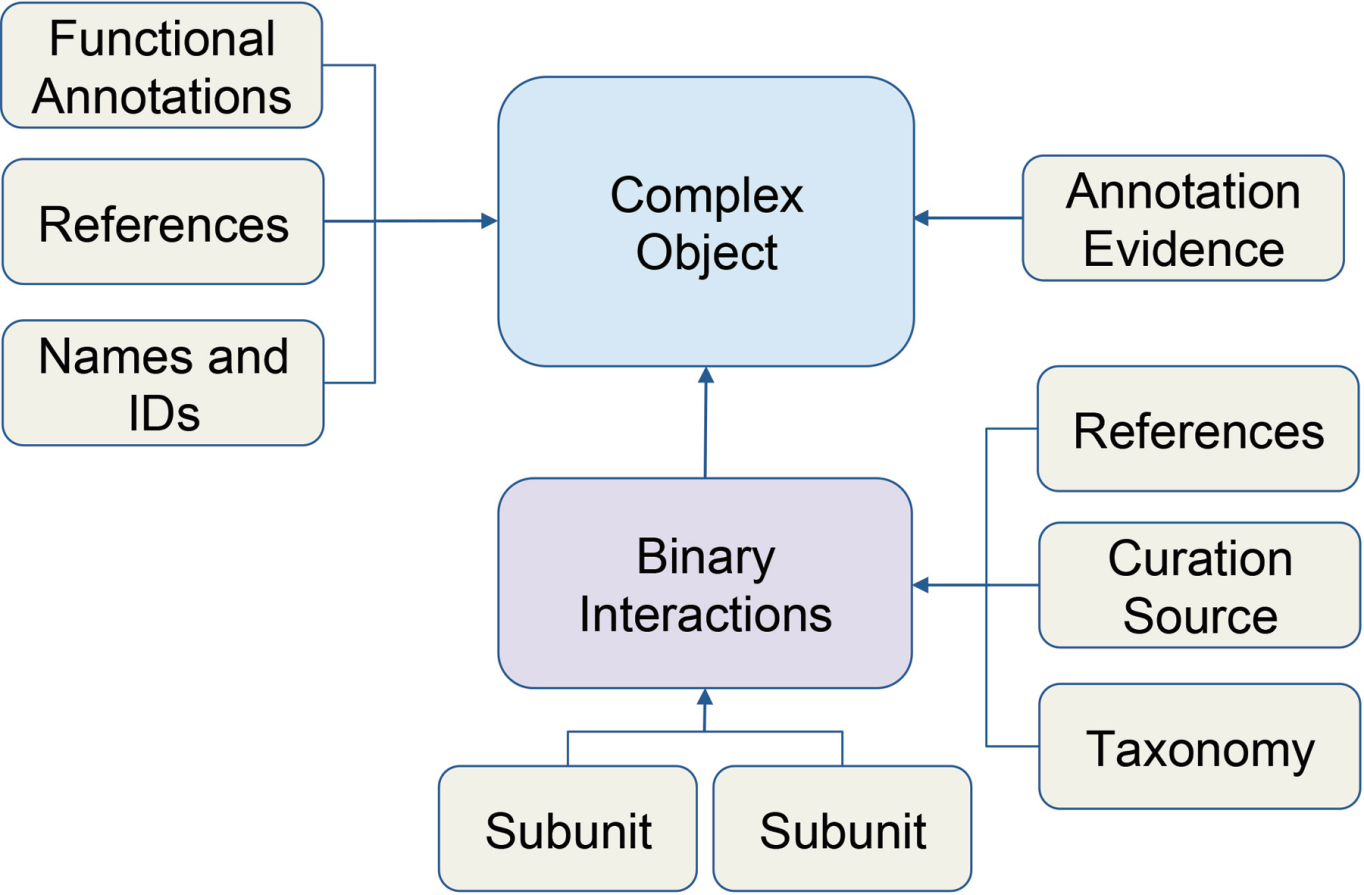
Schematic of macromolecular complex data object. Data for macromolecular complexes were expertly collected and curated from published literature. Each box represents a data type stored in the database. Arrows indicate how data is related to the complex object.

Complexes are stable entities that can be comprised of two or more interactors, which could be proteins, chemicals or other small molecules that can be isolated and shown to function together *in vivo.* Although each subunit may have a specific individual function, taken as a whole, these entities may have a different function altogether.

Biocurators from both groups collected the complex-relevant information from experimentally verified data published in peer-reviewed literature. Any integral non-protein molecules are also included in the complex. For each complex, biocurators record its composition (subunits), stoichiometry and topology. The Evidence and Conclusion Ontology (ECO; www.evidenceontology.org) is used to record the type of evidence available for each complex and experimental evidences are taken from IMEx member databases (5), Protein Data Bank (PDB; www.rcsb.org) (6) and The Electron Microscopy DataBank (EMDB; www.emdatabank.org) (7). Each complex is assigned a recommended name and a systematic name based on the protein participants (e.g. CCS1:SOD1). All common names (e.g. SOD1-CCS1 Superoxide Dismutase heterodimer) and synonyms used for that complex in the literature also are collected. Molecular function, biological process and cellular locations for the whole complex are curated using the GO vocabularies (www.geneontology.org) (8) and when available, cross-references to other databases, such as PDB for a complex’s physical structure or Reactome (www.reactome.org) (9) for a description of molecular pathways it acts in, are added. Short, free-text paragraphs summarize the function and properties of each curated complex. Literature references for all curated information are added to each entry. The data structure complies with the PSI-MI XML3.0 community standard (10). Our collaboration has resulted in the curation of the initial yeast macromolecular complexome, comprising of 589 macromolecular complexes (3). These data are stored at EMBL-EBI in the Complex Portal resource and have recently been integrated into the core database at SGD.

**Figure 2:**
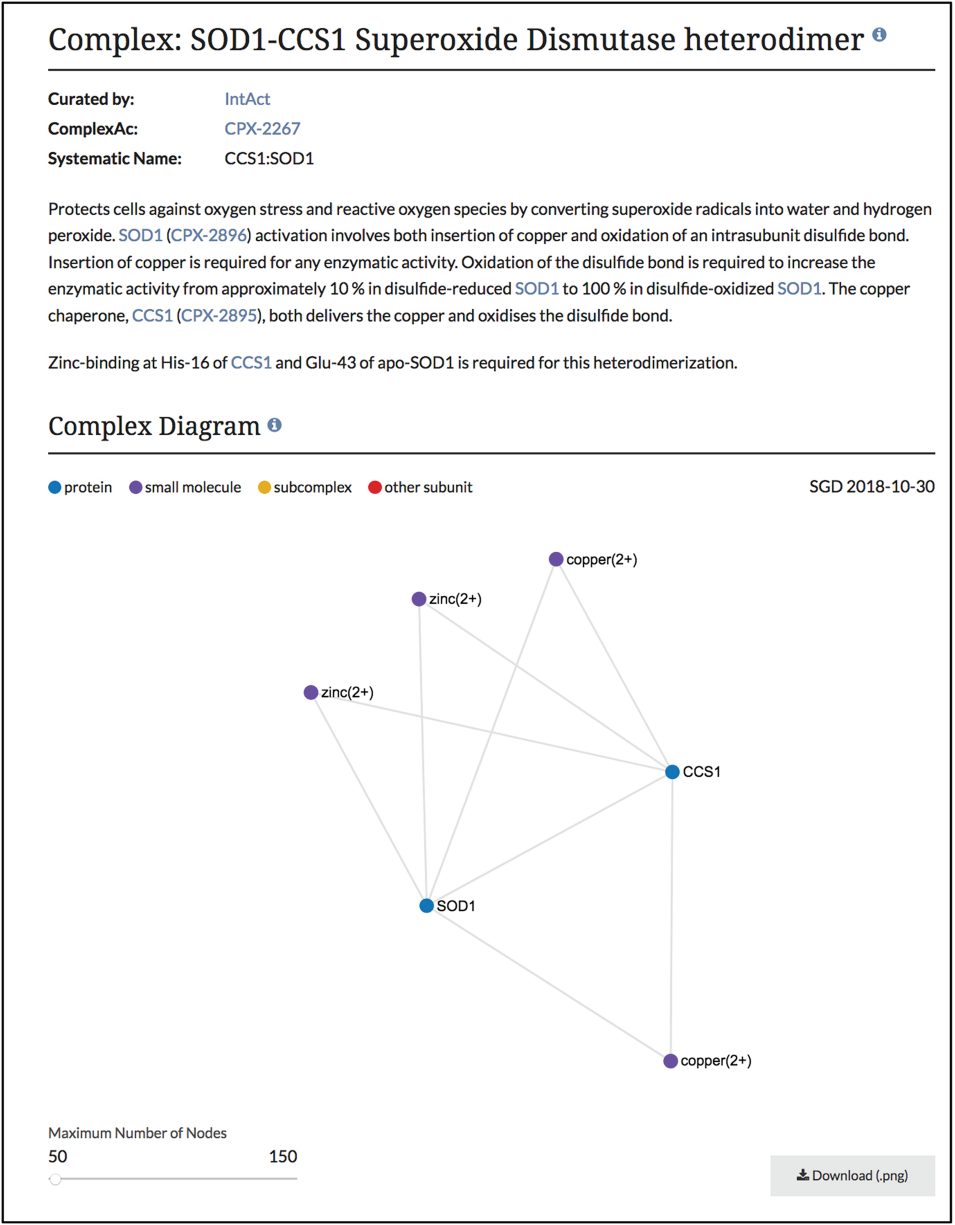
Summary and diagram sections of SOD1-CCS1 superoxide dismutase heterodimer page. Summary section that describes the function and composition of the SOD1-CCS1 superoxide dismutase complex. Individual interactions between subunits are shown in the Complex Diagram section. Complex diagrams can be downloaded as .png files. Complete page at www.yeastgenome.org/complex/CPX-2267.

**Figure 3:**
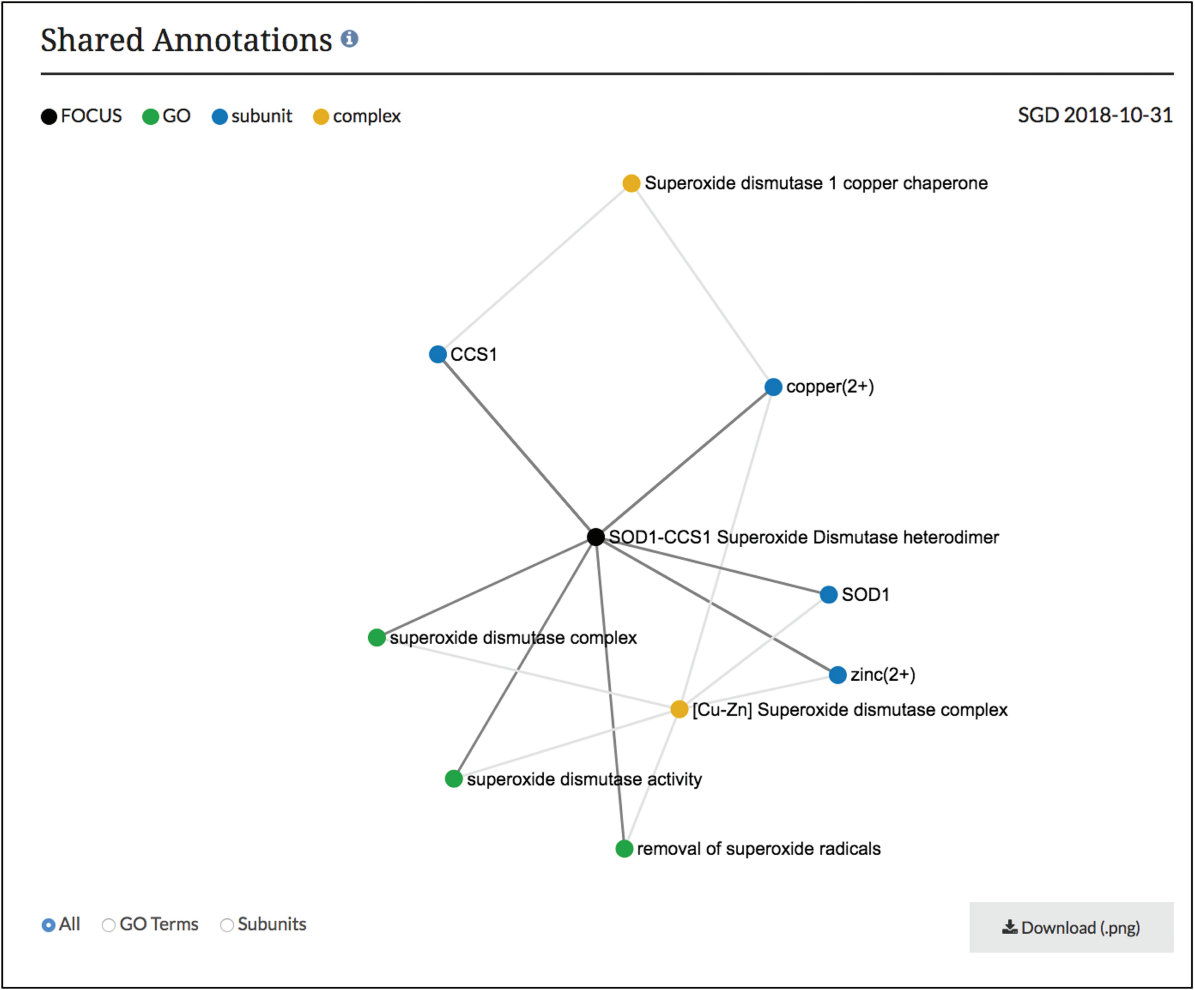
Network diagram of SOD1-CCS1 superoxide dismutase heterodimer page. GO annotations and complex subunits that are shared between the focus complex and other complexes are shown as a network. Users can select to see shared GO terms or shared subunits individually or both. Images can be downloaded as .png files.

## Integration of complex data into SGD

We added macromolecular complex data to YeastMine (yeastmine.yeastgenome.org), our data warehouse, in 2015, using the JAMI (Java Molecular Interaction framework) library to read molecular interaction data in PSI-MI XML3.0 format and translate these into interaction objects (11). We then created pre-generated queries and a list of all curated molecular complexes (yeastmine.yeastgenome.org/yeastmine/bagDetails.do?scope=all&bagName=All+Curated+MolecularComplexes) to allow SGD users to search for and explore the information associated with macromolecular complexes. Using the pre-generated queries, found under the ‘Interactions’ category, users can use genes to search for associated complexes (yeastmine.yeastgenome.org/yeastmine/template.do?name=Gene_Complex_New&scope=global) or use complex names to search for subunits and information (yeastmine.yeastgenome.org/yeastmine/template.do?name=Complex_Participant_Details&scope=global). Initial storage of the data in YeastMine allowed users quick access to these data while we were incorporating them into our core database.

We recently added these data into our core database using the Complex Portal JSON web services to retrieve data about individual complexes (e.g. www.ebi.ac.uk/intact/complex-ws/complex/CPX-2267). We store the macromolecular complexes as primary objects, along with its systematic name, Complex Portal ID, summary paragraphs, evidence annotation and reference information (Figure 1). All binary interactions between subunits of a complex are stored separately, along with stoichiometry, binding and reference details. Ontology terms, used to describe function, cellular location and annotation evidence among other details, are stored in separate ontology tables (e.g. ECO, GO, PSI-MI) and linked directly to macromolecular complex objects.

## Accessing macromolecular complex data at SGD

Using this information in our core database, SGD users can now find these macromolecular complex pages using our faceted search (www.yeastgenome.org/search?category=complex&page=0). Each page displays the summary paragraph about the complex’s composition and function, along with the group who did the curation of the complex (Figure 2). In addition, we have linkouts to the Complex Portal as well as to cross-references in other databases. The stoichiometry and binding information of the interacting molecules has been used to display a schematic of the complex and can also be found in a table, with descriptions of protein subunits. If available, images of the 3D structure created by RCSB PDB (www.rcsb.org) are also displayed, with links back to the original image and information at RCSB PDB (6). The function and cellular location of the entire macromolecular complex, not individual subunits, as annotated using GO terms, is also displayed on this page. While these pages share information and some displays with the Complex Portal pages, we added an additional network graph to facilitate discovery for researchers (Figure 3). This network graph displays GO annotations and interactors that are shared between whole complexes. Users have the option to visualize only shared GO annotations or only shared interactors, and by clicking on a term or complex, users can explore the information about each item.

## 

From YeastMine, these data are downloadable as tab- or comma-delimited text files, XML or JSON formats, either as a complete set or by focused lists of specific complexes as well as through the YeastMine API (yeastmine.yeastgenome.org/yeastmine/api.do). Additionally, data can be downloaded from SGD’s web services (e.g. www.yeastgenome.org/webservice/complex/CPX-2267). Example scripts demonstrating access our Application programming interface (API) are available on GitHub (https://github.com/yeastgenome/sgd_api_examples). All data are also accessible via the Complex Portal download page in PSI-MI XML 2.5 and 3.0, MI-JSON and ComplexTab formats (www.ebi.ac.uk/complexportal/download).

## Future directions

We will continue to collaborate with the Complex Portal curation team by updating information about previously curated macromolecular complexes, as well as curating any novel complexes as their discovery is reported in the literature. We will now be able to use these macromolecular complexes to expand the curation of pathways to further the understanding of cellular processes. This is an active collaboration between SGD and the Complex Portal to guarantee the most up-to-date information on molecular complexes are available to the scientific community.

## Notes

**Database URL:** www.yeastgenome.org
